# Tanshinone IIA Attenuates Renal Fibrosis after Acute Kidney Injury in a Mouse Model through Inhibition of Fibrocytes Recruitment

**DOI:** 10.1155/2015/867140

**Published:** 2015-12-29

**Authors:** Chunming Jiang, Qiuyuan Shao, Bo Jin, Rujun Gong, Miao Zhang, Biao Xu

**Affiliations:** ^1^Department of Nephrology, Affiliated Nanjing Drum Tower Hospital, Medical School of Nanjing University, Nanjing 21008, China; ^2^Department of Medicine, Rhode Island Hospital, Brown University School of Medicine, Providence, RI 02903, USA; ^3^Department of Cardiology, Affiliated Nanjing Drum Tower Hospital, Medical School of Nanjing University, Nanjing 21008, China

## Abstract

Acute kidney injury (AKI) is associated with an increased risk of developing advanced chronic kidney disease (CKD). Yet, effective interventions to prevent this conversion are unavailable for clinical practice. In this study, we examined the beneficial effects of Tanshinone IIA on renal fibrosis in a mouse model of folic acid induced AKI. We found that Tanshinone IIA treatment significantly attenuated the folic acid elicited kidney dysfunction on days 3, 14, and 28. This effect was concomitant with a much lessened accumulation of fibronectin and collagen in tubulointerstitium 28 days after folic acid injury, denoting an ameliorated renal fibrosis. The kidney protective and antifibrotic effect of Tanshinone IIA was likely attributable to an early inhibition of renal recruitment of fibrocytes positive for both CD45 and collagen I. Mechanistically, Tanshinone IIA treatment not only markedly diminished renal expression of chemoattractants for fibrocytes such as TGF*β*1 and MCP-1, but also significantly reduced circulating fibrocytes at the acute phase of kidney injury. These data suggested that Tanshinone IIA might be a novel therapy for preventing progression of CKD after AKI.

## 1. Introduction

Acute kidney injury (AKI) is a severe and common disease found in clinical practice. Recent data demonstrated that AKI occurs in about 3.2–9.6% of hospital admissions with overall mortality around 20% [1, 2]. Notably, AKI has been projected to be more prevalent around the world in the future due to the increasing incidence of hypertension, diabetes mellitus, caloric nutritional overload, and aging [3]. By far, a growing body of evidence has shown a close connection between AKI and subsequent chronic kidney disease (CKD), which substantially increases the long-term morbidity and mortality of AKI [4, 5]. Because effective treatments for AKI are unavailable to date except for supportive measures, it is imperative to develop a new therapeutic strategy to prevent AKI to CKD transition [6].

Enormous efforts have been dedicated to the understanding of the underlying pathophysiology of AKI; nevertheless, the exact mechanism contributing to AKI to CKD transition has not been fully elucidated. Collagen-producing hematopoietic cells (fibrocytes), first reported in 1994 by Bucala et al., possess both characteristics of fibroblasts (expression of collagen type I) and hematopoietic cells (expression of CD45) [7]. Recent evidence indicates that fibrocytes play an important role in promoting tissue fibrosis in various diseases including CKD [8–11]. During the process of tissue injury, fibrocytes have been found to undergo a rapid proliferation in the bone marrow and then were released into the peripheral circulation and finally recruited into the injury sites. It is well recognized that the migration of fibrocytes is mainly determined by chemoattractants due to their high expression of chemokine receptors and quick presentation in the inflammatory areas after injury. Up to now, several chemoattractants, such as transforming growth factor-beta (TGF-*β*), chemokines including monocyte chemotactic protein 1 (MCP-1), and lipopolysaccharide, which mediate the recruitment of fibrocytes have been identified [12–15].

Tanshinone IIA is a diterpene extracted from* Salvia miltiorrhiza*, a popular herb that has been safely and widely used in China and other Asian countries. Previous studies have demonstrated that Tanshinone IIA exerts prominent antifibrotic effect through modulating TGF-*β* signaling pathway and anti-inflammatory effect through inhibiting MCP-1 expression [16–24]. Thus, we hypothesized that Tanshinone IIA might attenuate renal fibrosis after AKI via regulating recruitment of fibrocytes into the kidney through its inhibition on TGF-*β* and MCP-1 in a mouse model of folic acid induced kidney injury.

## 2. Materials and Methods

### 2.1. Chemicals and Reagents

**Figure 1:**
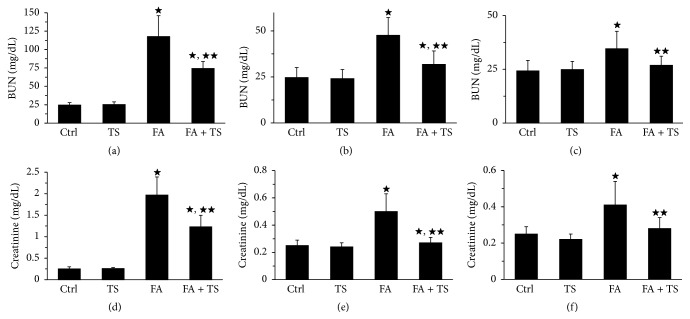
Tanshinone IIA ameliorates renal dysfunction in mice with folic acid induced acute kidney injury. Graphs show the time course of BUN and serum creatinine levels at day 3 (a, d), day 14 (b, e), and day 28 (c, f) among different groups (*n* = 9). Data are expressed as the mean ± SEM. ^★^ *P* < 0.01 versus group TS; ^★★^ *P* < 0.01 versus group FA. Notes: blood urea nitrogen (BUN). Ctrl: mice treated with vehicle alone; TS: mice treated with Tanshinone IIA alone; FA: folic acid-treated mice followed by vehicle treatment; FA + TS: folic acid-treated mice subjected to Tanshinone IIA injection for 3 consecutive days.

Tanshinone IIA was purchased from Jiangsu Carefree Group Co. (Nanjing, China). The structure of Tanshinone IIA is shown in Figure 1. Antibodies against fibronectin (ab2413), collagen I (ab34710), CD45 (ab25386), TGF-*β*1 (ab66043), and MCP-1 (ab25124) were obtained from Abcam, USA. Antibody against GAPDH (sc-48166) was obtained from Santa Cruz Biotechnology, USA. Both FITC- and HRP-conjugated secondary antibodies were purchased from Invitrogen, USA. ImmPRESS peroxidase polymer detection kits, DAB substrate (SK-4100), and Vectashield mounting medium with DAPI (H-1200) were obtained from Vector Laboratories, USA. BUN and creatinine assay kits were obtained from BioVision, USA. Hydroxyproline Assay Kit (MAK008), collagenase IV, and DNase I were purchased from Sigma, USA.

### 2.2. Animals

C57BL/6 mice weighing 180–220 g were obtained from Nanjing University Model Animal Research Center (Nanjing, China). The animals were housed in the animal facility of Nanjing University Model Animal Research Center with free access to food and water. The Institutional Animal Care and Use Committee at Drum Tower Hospital, the Affiliated Hospital of Nanjing University Medical School, approved all the animal protocols. The experiments were performed in accordance with the National Institutes of Health Guidelines on the Use of Laboratory Animals.

### 2.3. Experimental Procedures

C57BL/6 mice were randomly assigned to one of the four groups. In group Ctrl (*n* = 18), mice received 150 *μ*L saline with 0.02% DMSO by tail vein injection; in group TS (*n* = 18), mice received Tanshinone IIA (15 mg/kg, dissolved in 150 *μ*L saline with 0.02% DMSO) by tail vein injection; in group FA (*n* = 18), mice were injured with a single dose of folic acid (250 mg/kg, dissolved in 150 *μ*L sodium bicarbonate) by intraperitoneal injection and treated with 150 *μ*L saline with 0.02% DMSO by tail vein injection immediately after and on 2 other consecutive days following folic acid injection; in group FA + TS (*n* = 18), mice were injured with a single dose of folic acid (250 mg/kg) by intraperitoneal injection and treated with Tanshinone IIA (15 mg/kg) by tail vein injection immediately after and on 2 other consecutive days following folic acid injection. Mice were sacrificed at day 3 (*n* = 9 per group) or 28 (*n* = 9 per group) and kidney specimens were collected for further examinations. Blood samples were collected at days 3, 14, and 28 for detection of BUN and creatinine levels.

### 2.4. Renal Histopathology

Formalin-fixed kidneys were embedded in paraffin and prepared in 3 *μ*m thick sections. Sections were stained with Masson's trichrome to evaluate collagen deposition in the kidney. One observer performed semiquantitative morphometric analysis in a blinded manner. Fibrosis score was accessed using semiquantitative measurements according to the proportion relative to the total section area and classified as follows: 0 (nil), 1 (<25%), 2 (25–50%), 3 (50–75%), and 4 (>75% of interstitium).

For immunostaining formalin-fixed sections, 3 *μ*m thick kidney sections were deparaffinized and rehydrated. After microwave antigen retrieval, sections were H_2_O_2_ quenched and blocked in 3% BSA at room temperature. Tissues were incubated overnight with antibodies against TGF-*β*1 at 1 : 400, MCP-1 at 1 : 200 dilution at 4°C. ImmPRESS peroxidase polymer detection kits were used to conjugate secondary antibody for 60 minutes at room temperature. Slides were finally developed with DAB substrate. Negative controls were obtained by replacing the primary antibody with normal rabbit serum. The positive signal of TGF-*β*1 and MCP-1 staining was calculated with Image-Pro Plus 6.0 software (Media Cybernetics, Bethesda, MD). Each score was determined by five random fields per section and five sections per kidney.

Frozen tissue samples were cut into 3 *μ*m thick sections. After fixation with 3.2% paraformaldehyde in PBS, sections were blocked for 30 minutes with 3% BSA and permeabilized with Triton X-100 for 15 minutes. Then, sections were incubated overnight at 4°C with antibodies against fibronectin at 1 : 150, CD45 at 1 : 200, or collagen I at 1 : 300 dilution; FITC-conjugated secondary antibody against various species was incubated for 60 minutes at room temperature appropriately. Finally, sections were mounted with Vectashield mounting medium with DAPI and visualized using a fluorescence microscope. The positive signal was calculated with Image-Pro Plus 6.0 software or by counting positive staining cells. Each score was determined by five random fields per section and five sections per kidney.

### 2.5. Measurement of Total Collagen Content in the Kidney

Hydroxyproline concentration was measured to determine the total collagen content in the kidney using the Hydroxyproline Assay Kit according to the manufacturer's instruction. In brief, half of the right kidney was homogenized using 100 *μ*L H_2_O for every 10 mg of tissue. Then, 100 *μ*L of tissue homogenate was transferred to a pressure-tight vial; 100 *μ*L concentrated hydrochloric acid (12 M) was added and hydrolyzed at 120°C for 3 h. Fifty *μ*L of supernatant was transferred to a 96-well plate. Evaporate all wells to dryness under vacuum. Add 100 *μ*L of the chloramine T/oxidation buffer mixture to each sample and standard well, incubated at room temperature for 5 minutes. Add 100 *μ*L of the diluted DMAB reagent to each sample and standard well, incubated for 90 minutes at 60°C. Absorbance was measured at 560 nm in a microplate reader.

### 2.6. Western Blot Analysis

Mice cortical kidney specimens were homogenized in radioimmunoprecipitation assay buffer supplemented with protease inhibitors. Protein concentration was determined by using a bicinchoninic acid protein assay kit. Samples with equal amounts of total protein (50 *μ*g/mL) were fractionated by 7.5–15% SDS-polyacrylamide gels under reduction. The antibodies against fibronectin, TGF-*β*1, MCP-1, and GAPDH were used as primary antibodies.

### 2.7. Measurement of Blood Urea Nitrogen (BUN) and Serum Creatinine

BUN and serum creatinine levels were measured using commercial assay kits according to the manufacturer's instruction.

### 2.8. Statistical Analysis

Normally distributed continuous variables are expressed as mean ± standard error. One-way analysis of variance was applied to compare the means of normally distributed continuous variables followed by multiple comparison tests as post hoc comparison. Mann-Whitney test or Kruskal-Wallis test was applied to compare the abnormally distributed continuous variables as appropriate. A value of *p* < 0.05 was considered statistically significant, and all tests were two-tailed. All statistical analyses were performed with the SPSS software application (version 17.0 for Windows: SPSS Institute, Chicago, IL, USA).

## 3. Result

### 3.1. Tanshinone IIA Treatment Improves Renal Function following Folic Acid Injury

The time-course change of renal function among different animal groups was shown in Figure 1. BUN and serum creatinine levels were markedly elevated on day 3 after folic acid injection and this was significantly lowered by concomitant Tanshinone IIA treatment (Figures 1(a) and 1(d)). Three days after folic acid injury, mice in group FA exhibited spontaneous recovery of kidney function, as shown by the decreased levels of BUN and serum creatinine on days 7 and 28. However, this recovery of kidney function was apparently incomplete, as evidenced by higher-than-normal levels of BUN and serum creatinine on day 28 in group FA (Figures 1(b), 1(c), 1(e), and 1(f)). In contrast, in mice treated with Tanshinone IIA, BUN and serum creatinine levels were almost fully restored at day 28 after folic acid exposure. These data indicate that Tanshinone IIA can effectively protect from kidney dysfunction elicited by folic acid injury.

### 3.2. Tanshinone IIA Ameliorates the Folic Acid Induced Kidney Fibrosis

**Figure 2:**
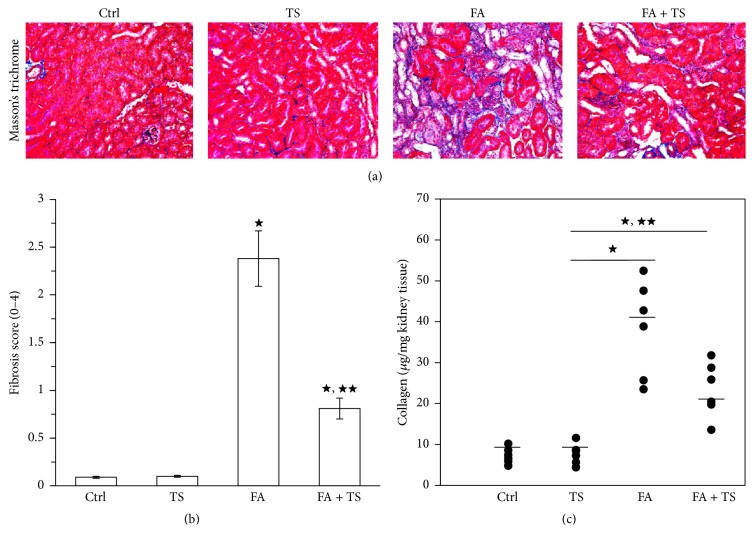
Tanshinone IIA reduces collagen deposition in the kidney 28 days after folic acid injury. (a) Representative Masson-stained sections of renal cortices at day 28 (original magnification ×200). (b) Semiquantification of kidney fibrosis score from Masson-stained sections. (c) Total kidney collagen content determined by hydroxyproline detection among different groups (*n* = 6). Data are expressed as the mean ± SEM (*n* = 6 or 9). ^★^ *P* < 0.01 versus group TS; ^★★^ *P* < 0.01 versus group FA. Notes: Ctrl: mice treated with vehicle alone; TS: mice treated with Tanshinone IIA alone; FA: folic acid-treated mice followed by vehicle treatment; FA + TS: folic acid-treated mice subjected to Tanshinone IIA injection for 3 consecutive days.

**Figure 3:**
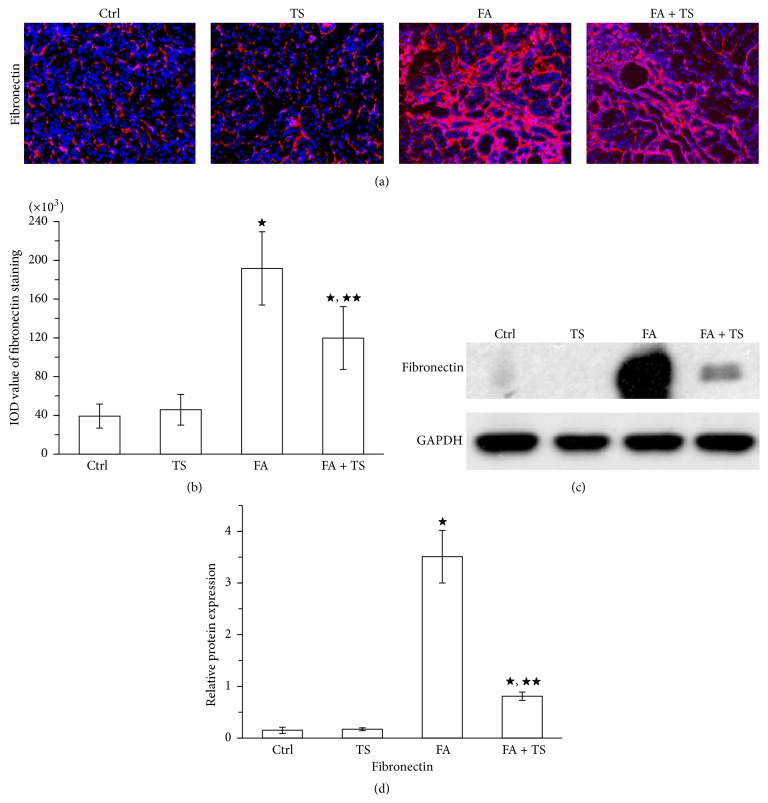
Tanshinone IIA inhibits fibronectin expression in the kidney 28 days after folic acid injury. (a) Representative images of fibronectin expression at day 28 stained by immunofluorescence (original magnification ×100). (b) Semiquantification of kidney fibronectin deposition from (a). (c) Representative immunoblots and semiquantification (d) of fibronectin in kidney cortical tissue lysates at day 28. Data are expressed as the mean ± SEM (*n* = 9). ^★^ *P* < 0.01 versus group TS; ^★★^ *P* < 0.01 versus group FA. Notes: Ctrl: mice treated with vehicle alone; TS: mice treated with Tanshinone IIA alone; FA: folic acid-treated mice followed by vehicle treatment; FA + TS: folic acid-treated mice subjected to Tanshinone IIA injection for 3 consecutive days.

Folic acid induced kidney injury can lead to progressive kidney fibrosis. In the present study, collagen deposition in the kidneys was assessed by Masson's trichrome staining. Folic acid insult resulted in a massive collagen deposition in renal tubulointerstitium on day 28 (Figures 2(a) and 2(b)). This was further confirmed by the detection of total collagen contents in the kidneys (Figure 2(c)). In accordance, increased accumulation of fibronectin in renal tubulointerstitium was also observed in group FA mice on day 28 (Figure 3). These fibrotic changes in the folic acid-injured kidneys were remarkably abrogated by Tanshinone IIA treatment, as shown by less collagen and fibronectin deposition in kidney sections. Collectively, these data suggest that Tanshinone IIA treatment at the early stage of AKI can improve the long-term outcome of the injured kidney by ameliorating kidney fibrosis.

### 3.3. Tanshinone IIA Reduces Renal Accumulation of Fibrocytes Early after Folic Acid Injury

**Figure 4:**
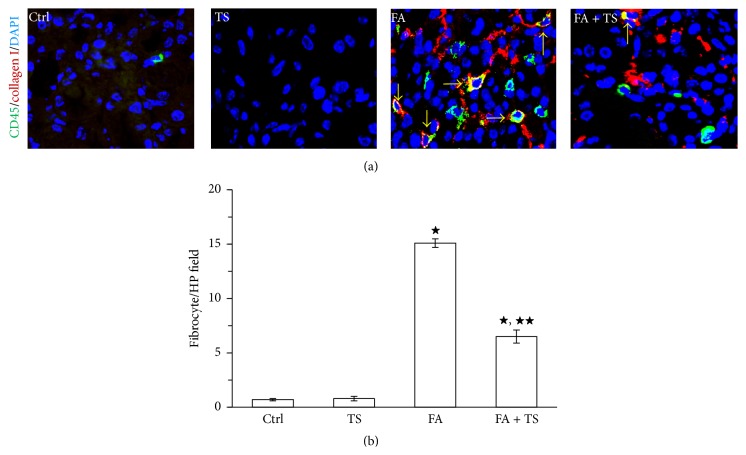
Tanshinone IIA inhibits recruitment of fibrocytes into the folic acid-injured kidney. (a) Representative images of fibrocytes in the kidney (yellow arrows), determined by dual staining of CD45 (green color) and collagen I (red color) in the kidney. (b) Quantification of fibrocytes in kidney sections evaluated in five random fields per section and five sections per kidney. Data are expressed as the mean ± SEM (*n* = 9). ^★^ *P* < 0.01 versus group TS; ^★★^ *P* < 0.01 versus group FA. Notes: Ctrl: mice treated with vehicle alone; TS: mice treated with Tanshinone IIA alone; FA: folic acid-treated mice followed by vehicle treatment; FA + TS: folic acid-treated mice subjected to Tanshinone IIA injection for 3 consecutive days.

Previously, fibrocytes have been demonstrated to contribute to kidney fibrosis after various chronic injuries. In the present study, we examined the influence of Tanshinone IIA on the accumulation of fibrocytes in folic acid-injured kidney on day 3. As shown in Figure 4, very few fibrocytes, determined by dual positive staining of CD45 and collagen I, were observed in the kidney sections from control or TS group mice. Administration of folic acid resulted in a significant increase in fibrocytes accumulation in the kidney on day 3. In contrast, treatment with Tanshinone IIA significantly decreased the numbers of fibrocytes presented in the sections. These data suggest that Tanshinone IIA attenuates folic acid induced kidney fibrosis at least partially by inhibiting accumulation of fibrocytes in the injured kidney.

### 3.4. Tanshinone IIA Decreases Renal Expression of TGF-*β*1 and MCP-1 Expression 3 Days after Folic Acid Injury

**Figure 5:**
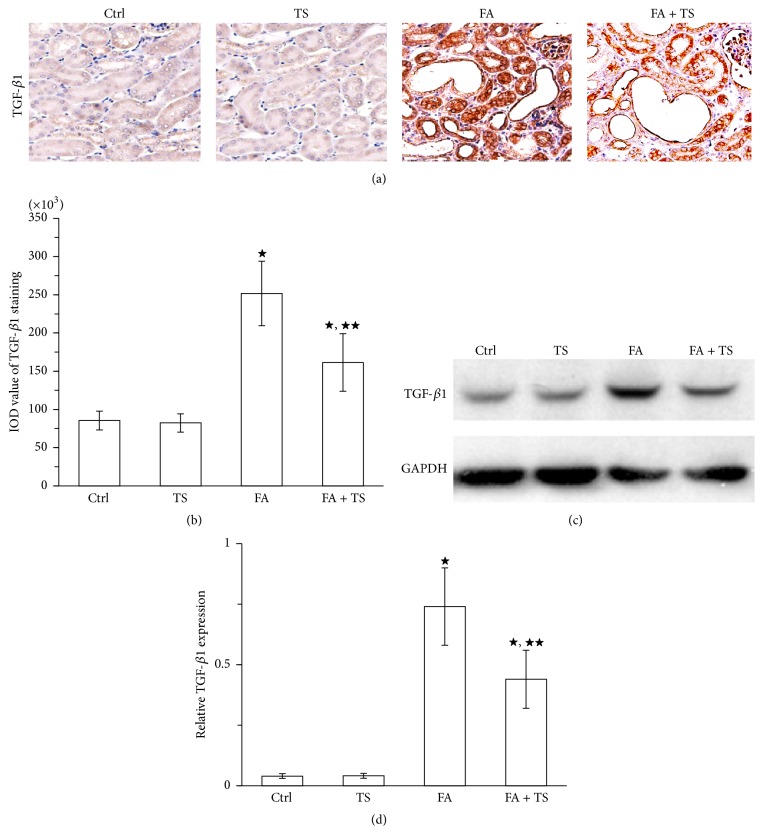
Tanshinone IIA inhibits TGF-*β*1 expression in folic acid-injured kidney at day 3. (a) Representative images and semiquantification (b) of TGF-*β*1 (original magnification ×400) stained by immunohistochemistry in the kidney sections at day 3. (c) Representative immunoblot and quantification (d) of TGF-*β*1 in kidney cortical tissue lysates at day 3. Data are expressed as the mean ± SEM (*n* = 9). ^★^ *P* < 0.01 versus group TS; ^★★^ *P* < 0.01 versus group FA. Notes: transforming growth factor-beta 1 (TGF-*β*1); Ctrl: mice treated with vehicle alone; TS: mice treated with Tanshinone IIA alone; FA: folic acid-treated mice followed by vehicle treatment; FA + TS: folic acid-treated mice subjected to Tanshinone IIA injection for 3 consecutive days.

**Figure 6:**
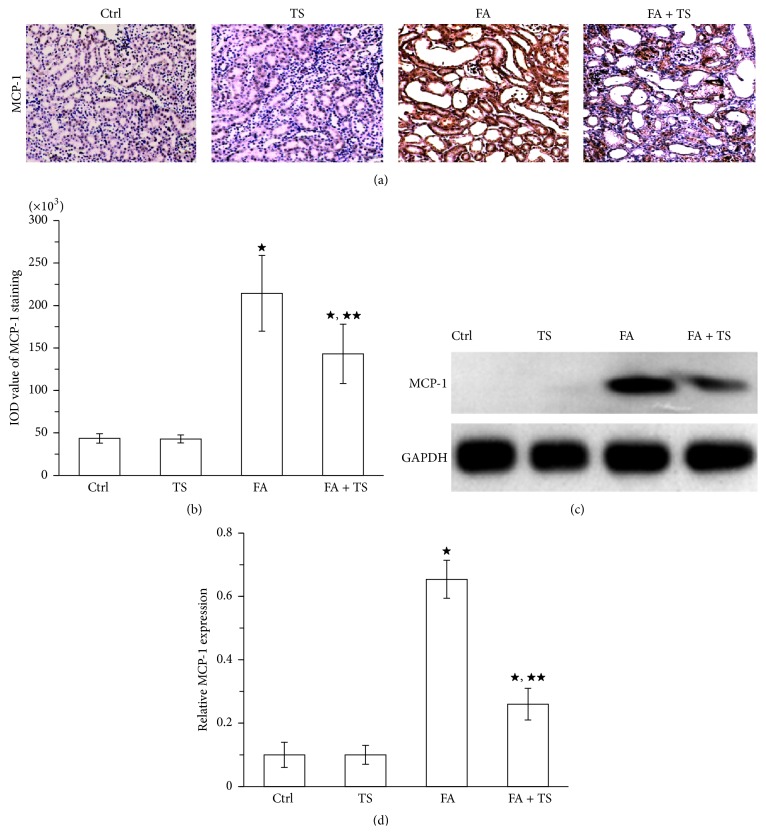
Tanshinone IIA inhibits MCP-1 expression in the folic acid-injured kidney at day 3. (a) Representative images and semiquantification (b) of MCP-1 (original magnification ×100) stained by immunohistochemistry in the kidney sections at day 3. (c) Representative immunoblot and quantification (d) of MCP-1 in kidney cortical tissue lysates at day 3. Data are expressed as the mean ± SEM (*n* = 9). ^★^ *P* < 0.01 versus group TS; ^★★^ *P* < 0.01 versus group FA. Notes: monocyte chemotactic protein 1 (MCP-1); Ctrl: mice treated with vehicle alone; TS: mice treated with Tanshinone IIA alone; FA: folic acid-treated mice followed by vehicle treatment; FA + TS: folic acid-treated mice subjected to Tanshinone IIA injection for 3 consecutive days.

Both TGF-*β*1 and MCP-1 are important chemoattractants for fibrocytes and play critical roles in recruitment of fibrocytes into the injured tissues. In present study, we measured their expression by peroxidase immunohistochemistry staining and immunoblot analysis in the kidney 3 days after folic acid exposure. As shown in Figures 5(a), 5(b), 6(a), and 6(b), administration of folic acid resulted in a significant increase of TGF-*β*1 and MCP-1 expression in the kidney. In contrast, Tanshinone IIA treatment largely suppressed their expression. This was further confirmed by immunoblot analysis of kidney cortical lysates for TGF-*β*1 and MCP-1 (Figures 5(c), 5(d), 6(c), and 6(d)). These data suggest that Tanshinone IIA may also reduce recruitment of fibrocytes into the kidney by inhibiting TGF-*β*1 and MCP-1 expression in the injured kidney.

## 4. Discussion

In this study, we demonstrate that Tanshinone IIA treatment exerts beneficial effects on renal fibrosis of a mouse model of folic acid induced AKI. Our results showed that the accumulation of fibrocytes in the kidney was evidently suppressed by Tanshinone IIA treatment at the early stage of AKI. The possible underlying mechanisms might be its inhibition of renal expression of TGF-*β*1 and MCP-1, key chemoattractants contributing to the fibrocytes recruitment into the injured kidney. These findings indicate that Tanshinone IIA is a potential agent to prevent progression of CKD after AKI.

AKI is a severe disease which is increasingly prevalent around the world. Despite the fact that most AKI exhibit clinically spontaneous recovery, recent studies demonstrate that they are responsible for the development of subsequent CKD. However, effective therapies for preventing CKD after AKI are still scarce. Tanshinone IIA has been demonstrated to possess remarkable antifibrosis properties on various tissues or organs including peritoneum, heart, lung, and liver [16, 19–22, 25–27]. Notably, the effect of Tanshinone IIA on preventing renal fibrosis has also been proved. For example, Tanshinone IIA can attenuate renal fibrosis in a rat model of 5/6 nephrectomy [28]. Tanshinone IIA has also been shown to inhibit collagen deposition on a rat model of diabetic nephropathy [29]. In the present study, we explored the effect of Tanshinone IIA on preventing renal fibrosis after AKI and found that it can effectively improve kidney function and attenuate histological fibrosis 28 days after folic acid exposure. This result indicates that Tanshinone IIA is a potent agent for preventing renal fibrosis after AKI.

Despite the fact that the precise mechanisms by which renal fibrosis occurred after AKI are not clear yet, fibrocytes have attracted much attention for their role in tissue repair and fibrosis via various mechanisms. A recent study showed that the depletion of fibrocytes by diphtheria toxin resulted in a significant reduction of collagen deposition in the obstructed mice kidney [11]. In the present study, we found that Tanshinone IIA significantly reduced the accumulation of fibrocytes in the kidney 3 days after folic acid insult. This finding suggests that interception of the recruitment of fibrocytes into the injured kidney might be an important mechanism underlying the antifibrotic effects of Tanshinone IIA. To our knowledge, this is the first study to show the beneficial effects of Tanshinone IIA on preventing renal fibrosis via inhibiting accumulation of fibrocytes in the kidney.

How Tanshinone IIA intercepts renal recruitment of fibrocytes, however, is still unknown. TGF-*β*1 is a well-recognized growth factor that causes fibrosis. TGF-*β*1 can promote fibrosis by enhancing the synthesis and by suppressing the degradation of extracellular matrix. Moreover, recent studies have also demonstrated a role of TGF-*β*1 in chemoattracting fibrocytes into various injured organs [12, 14, 15, 30]. For example, infection of mice with TGF-*β*1 expressing adenoviral vector resulted in a rapid recruitment of fibrocytes to the liver [31]. Similarly, TGF-*β*1 also evidently triggered fibrocyte mobilization into fibrotic lungs and kidneys [12, 13]. Previous studies have clearly demonstrated that Tanshinone IIA can inhibit TGF-*β*1 expression in many organs including the heart, kidney, and lung [18, 32, 33]. In agreement, our recent data also revealed that Tanshinone IIA could attenuate peritoneal fibrosis by suppressing TGF*β*1 expression [22]. Again, in the present study, we confirmed the effect of Tanshinone IIA on inhibiting TGF-*β*1 in folic acid induced kidney injury. Our data indicated the role of Tanshinone IIA in preventing recruitment of fibrocytes into the kidney by reducing TGF-*β*1 expression.

In addition to TGF-*β*1, chemokines also play important roles in mediating the homing of fibrocytes to fibrotic lesions [8, 34]. Previous studies demonstrated that MCP-1 signaling pathway regulates fibrocytes migration into the kidney and evidently contributed to the renal fibrosis [35, 36]. Recently, Tanshinone IIA has been found to reduce MCP-1 expression in various organs, including cardiac tissues subjected to myocardial infarction and kidneys after 5/6 nephrectomy or subjected to uric acid injury [24, 37]. Consistently, we demonstrated in this study that Tanshinone IIA treatment significantly attenuated MCP-1 expression in the folic acid induced kidney injury, suggesting that Tanshinone IIA may inhibit the migration of fibrocytes into the injured kidney by downregulating MCP-1 expression. Moreover, Tanshinone IIA was likely effective in reducing the amount of fibrocytes after kidney injury.

In addition to the above-mentioned mechanism, other potential possibilities contributing to the decrease of fibrocytes in the injured kidney by Tanshinone IIA need further study. For example, in vitro experiments are helpful to address whether Tanshinone IIA could directly modulate fibrocytes differentiation for we found that Tanshinone IIA significantly reduced circulating fibrocytes on day 3 after folic acid administration (data shown in Supplementary Material available online at http://dx.doi.org/10.1155/2015/867140). Moreover, the mechanism responsible for AKI to CKD transition is complicated and far from being elucidated. By far, several factors including the severity of AKI, maladaptive repair of injured renal cells, and unresolved inflammation are the main suspected culprits for CKD development after AKI. As implied by other studies, Tanshinone IIA might possess activities on reducing tissue damage, promoting cell recovery, and inhibiting inflammation response during the acute phase of organ injury. Therefore, our study does not exclude other potential possibilities that could contribute to antifibrotic effect of Tanshinone IIA.

## 5. Conclusion

Tanshinone IIA significantly attenuated the folic acid induced kidney fibrosis by inhibiting the recruitment of fibrocytes into the kidney. This action of Tanshinone IIA is at least partially associated with its inhibitory effect on TGF-*β*1 and MCP-1 expression, important chemoattractants for fibrocytes recruitment into the kidney. Our findings suggest that Tanshinone IIA might be a potential agent to be applied in clinic for preventing progression of CKD after AKI. Further studies are needed to investigate its role in other models of kidney injury such as unilateral ureteral occlusion and ischemia-reperfusion injury.

## Supplementary Material

Flow cytometric analysis on circulating fibrocytes showed that the number of fibrocytes was significantly increased 3 days after folic acid injection. Tanshinone IIA administration drastically inhibited this effect of folic acid. Our data suggests that Tanshinone IIA may reduce the number of fibrocytes in the folic acid injured kidney by decreasing the amount of circulating fibrocytes.
