# Supplementary material for: Tanshinone IIA Attenuates Renal Fibrosis after Acute Kidney Injury in a Mouse Model through Inhibition of Fibrocytes Recruitment
Source: Biomed Res Int. 2015 Dec 29;2015:867140. doi: 10.1155/2015/867140 (PMC4739267; doi:10.1155/2015/867140)
Supplement: Supplementary file 1 — Flow cytometric analysis on circulating fibrocytes showed that the number of fibrocytes was significantly increased 3 days after folic acid injection. Tanshinone IIA administration drastically inhibited this effect of folic acid. Our data suggests that Tanshinone IIA may reduce the number of fibrocytes in the folic acid injured kidney by decreasing the amount of circulating fibrocytes. [file 867140.f1.docx]

**Supplementary material**

**Concise method and result**

**Method**

In order to evaluate the effect of Tanshinone IIA on fibrocytes in the circulation, 9 C57BL/6 mice were randomly assigned to the Ctrl; FA and FA+TS groups. At day 3, blood sample was collected in the presence of 500 mMEDTA from the inferior vena cava. Red blood cells were firstly lysed with Red Blood Cell Lysis Buffer. Then, surface antigens were labeled with PE-labeled anti- and FITC-labeled rabbit anti-collagen I antibodies at 1:250 dilution. Antibodies and their isotype controls were used according to the manufacturer’s recommendations. Data were collected on a FACSArray flow cytometer (BD Biosciences) and analyzed using FlowJo software (TreeStar, Ashland, OR, USA).

**Result**

As shown in Figure S1, 3 days after folic acid injection, the number of fibrocytes in the circulation was significantly increased as compared with that in the Ctrl group. Tanshinone IIA drastically inhibited this effect of folic acid, suggesting that the Tanshinone IIA induced reduction of fibrocytes in the folic acid injured kidney may be partially attributable to its effect on decreasing the amount of circulating fibrocytes.


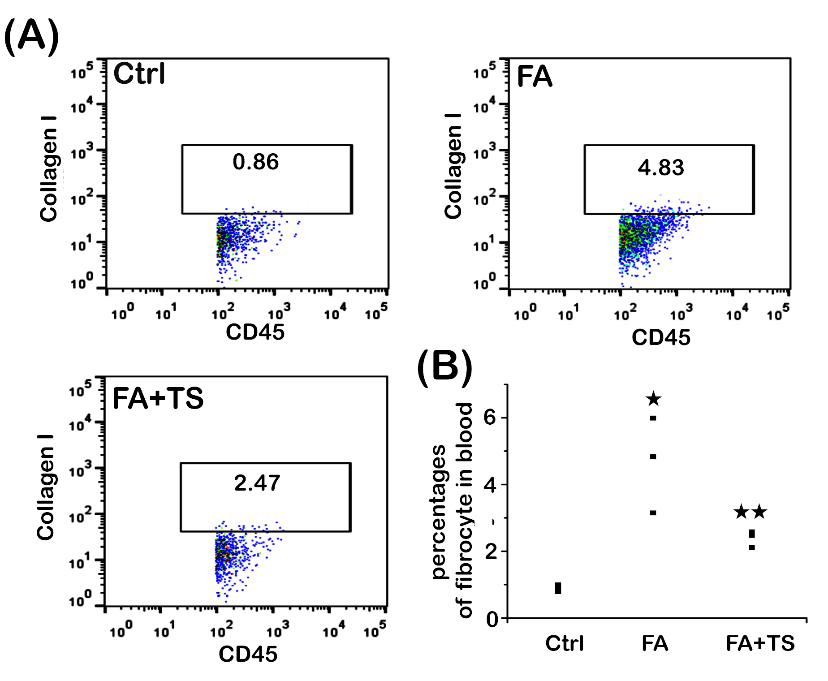


**FIGURE S1: Tanshinone IIA decreases the number of fibrocytes 3 days after folic**

**acid injury.** (A) Representative dot plots are shown for the ratio of fibrocytes

(CD45+Collagen I+) versus CD45+ cells in the blood; cells were gated on CD45+ cells. (B)

The percentage of fibrocytes of each experimental group was measured (n=3). * P<0.01 vs.

group Ctrl; **P<0.05 vs. group FA. Notes: Ctrl, mice treated with vehicle alone; FA, folic

acid-treated mice followed by vehicle treatment; FA+TS, folic acid-treated mice subjected

to Tanshinone IIA injection for 3 consecutive days.
